# The *Arabidopsis thaliana* Knockout Mutant for *Phytochelatin Synthase1* (*cad1-3*) Is Defective in Callose Deposition, Bacterial Pathogen Defense and Auxin Content, But Shows an Increased Stem Lignification

**DOI:** 10.3389/fpls.2018.00019

**Published:** 2018-01-22

**Authors:** Maria De Benedictis, Cecilia Brunetti, Elizabeth K. Brauer, Andrea Andreucci, Sorina C. Popescu, Mauro Commisso, Flavia Guzzo, Adriano Sofo, Monica Ruffini Castiglione, Olena K. Vatamaniuk, Luigi Sanità di Toppi

**Affiliations:** ^1^Department of Life Sciences, University of Parma, Parma, Italy; ^2^Soil and Crop Sciences Section, School of Integrative Plant Sciences, Cornell University, Ithaca, NY, United States; ^3^National Research Council of Italy, Istituto Per La Valorizzazione Del Legno E Delle Specie Arboree, Florence, Italy; ^4^Agriculture and Agri-Food Canada, Ottawa, ON, Canada; ^5^Department of Biology, University of Pisa, Pisa, Italy; ^6^Department of Biochemistry, Molecular Biology, Entomology, and Plant Pathology, Mississippi State University, Starkville, MS, United States; ^7^Department of Biotechnology, University of Verona, Verona, Italy; ^8^School of Agricultural, Forestry, Food and Environmental Sciences, University of Basilicata, Potenza, Italy

**Keywords:** auxin, cadmium, flavonol, glucosinolate, phenylpropanoid, phytochelatin synthase

## Abstract

The enzyme phytochelatin synthase (PCS) has long been studied with regard to its role in metal(loid) detoxification in several organisms, i.e., plants, yeasts, and nematodes. It is in fact widely recognized that PCS detoxifies a number of heavy metals by catalyzing the formation of thiol-rich oligomers, namely phytochelatins, from glutathione and related peptides. However, recent investigations have highlighted other possible roles played by the PCS enzyme in the plant cell, e.g., the control of pathogen-triggered callose deposition. In order to examine novel aspects of *Arabidopsis thaliana* PCS1 (AtPCS1) functions and to elucidate its possible roles in the secondary metabolism, metabolomic data of *A. thaliana* wild-type and *cad1-3* mutant were compared, the latter lacking AtPCS1. HPLC-ESI-MS analysis showed differences in the relative levels of metabolites from the glucosinolate and phenylpropanoid pathways between *cad1-3* and wild-type plants. Specifically, in control (Cd-untreated) plants, higher levels of 4-methoxy-indol-3-ylmethylglucosinolate were found in *cad1-3* plants vs. wild-type. Moreover, the *cad1-3* mutant showed to be impaired in the deposit of callose after Cd exposure, suggesting that AtPCS1 protects the plant against the toxicity of heavy metals not only by synthesizing PCs, but also by contributing to callose deposition. In line with the contribution of callose in counteracting Cd toxicity, we found that another callose-defective mutant, *pen2-1*, was more sensitive to high concentrations of Cd than wild-type plants. Moreover, *cad1-3* plants were more susceptible than wild-type to the hemibiotrophic bacterial pathogen *Pseudomonas syringae*. The metabolome also revealed differences in the relative levels of hydroxycinnamic acids and flavonols, with consequences on cell wall properties and auxin content, respectively. First, increased lignification in the *cad1-3* stems was found, probably aimed at counteracting the entry of Cd into the inner tissues. Second, in *cad1-3* shoots, increased relative levels of kaempferol 3,7 dirhamnoside and quercetin hexoside rhamnoside were detected. These flavonols are endogenous inhibitors of auxin transport *in planta*; auxin levels in both roots and shoots of the *cad1-3* mutant were in fact lower than those of the wild-type. Overall, our data highlight novel aspects of AtPCS1 functions in *A. thaliana*.

## Introduction

Phytochelatins (PCs) are thiol-rich oligopeptides that facilitate the sequestration in the vacuo-lysosomal compartments of several metal(loid)s (Cobbett, 2000; Mendoza-Cózatl et al., 2011; Rea, 2012). PCs are post-translationally synthesized by the enzyme PCS, a γ-glutamylcysteine dipeptidyl (trans)peptidase (EC 2.3.2.15) (Grill et al., 1989; Rauser, 1990; Zenk, 1996; Clemens et al., 1999; Ha et al., 1999; Vatamaniuk et al., 1999, 2000, 2004; Romanyuk et al., 2006; Rea, 2012). In *Arabidopsis thaliana* the transpeptidation reaction is performed by the AtPCS1 enzyme, characterized for the first time by Ha et al. (1999); indeed, the importance of AtPCS1 in metal detoxification was demonstrated by the *A. thaliana cad1-3* mutant, which lacks a functional PCS1, thus being PC-deficient and Cd-hypersensitive (Howden et al., 1995). Likewise, the PCS-deficient mutants of fission yeast *Schizosaccharomyces pombe* and of the nematode *Caenorhabditis elegans* are hypersensitive to heavy metals (Ha et al., 1999; Vatamaniuk et al., 2001). Thus, the PC synthesis via PCS activation is essential for metal(loid) detoxification in several organisms, including basal land plants (Degola et al., 2014; Petraglia et al., 2014).

Interestingly, Clay et al. (2009) found a close correlation between AtPCS1 activity and indole glucosinolate biosynthesis. This class of secondary metabolites is constitutively synthesized in plants, predominantly in the Brassicaceae family (Fahey et al., 2001), and include: (1) aliphatic glucosinolates, derived primarily from methionine; (2) indole glucosinolates, derived from tryptophan; and (3) aromatic glucosinolates, from phenylalanine or tyrosine (for a review, see Halkier and Gershenzon, 2006). Glucosinolates are stored in the vacuole, and when the cell is damaged (e.g., by herbivores), they are released from this compartment and hydrolyzed by the endogenous enzyme myrosinase (β-thioglucosidase). The products of the glucosinolate-myrosinase system are biologically active molecules, which include isothiocyanates, nitriles, thiocyanates, oxazolidine-2-thiones, and epithionitriles, with multiple physiological functions as defense against herbivors, parasites and pathogens, and cell signaling (Wittstock and Halkier, 2002; Kim et al., 2008; Wittstock and Burow, 2010; Burow and Halkier, 2017).

In addition, in response to a pathogen or herbivore attack, plants can deposit callose [β(1,3)-glucan] into cell walls, that is a defense mechanism interpretable in terms of “Microbe-Triggered Immunity” (MTI) or, in sporadic cases, of “Herbivore-Triggered Immunity” (HTI) (Thordal-Christensen, 2003; Nürnberger and Lipka, 2005).

Unlike wild-type, the *cad1-3* mutant of *A. thaliana*, which lacks a functional AtPCS1 enzyme, is impaired in callose deposition after Flg22 treatments (Flg22 is a synthetic polypeptide that represents one of the highly conserved epitopes of the bacterial flagellin) (Clay et al., 2009). The *cad1-3* mutant also accumulates a callose precursor, the indole glucosinolate 4MOI3M (*sin.* 4-methoxy-I3G; Clay et al., 2009). Likewise, other mutants from the glucosinolate synthesis pathway, including *pen2-1, pen2-2, pen3-1*, etc., show an accumulation of 4MOI3M and defects in the callose response after Flg22 treatment (Clay et al., 2009; Luna et al., 2011). Transcriptional data of *PEN2, PEN3*, and *PCS1* genes suggest their mutual interaction aimed at the hydrolysis of 4MOI3M, with consequent callose deposition (Clay et al., 2009). Concerning this, PEN2 acts as a putative myrosinase enzyme (a type of β-thio-glucoside glycohydrolase), which hydrolyzes indole glucosinolates *in planta* (Lipka et al., 2005; Bednarek et al., 2009), and represents an important component in oomycete defense response (Schlaeppi and Mauch, 2010). PEN3 is an ABC transporter located on the plasma membrane, able to export Cd and IBA from the cell (Kim et al., 2007; Strader and Bartel, 2009). Its involvement in the movement of toxic metabolites toward pathogen penetration sites has been also hypothesized (Stein et al., 2006; Bednarek et al., 2009), since a higher accumulation of PEN3 has been found under fungal appressoria during infection (Stein et al., 2006).

Thus, considering that the *cad1-3* mutant is defective in callose deposition (Clay et al., 2009), AtPCS1 may possibly play a relevant role in the innate immune response, particularly as far as MTI is concerned. In line with this, the *cad1-3* mutant has been demonstrated to be more susceptible to the oomycete *Phytophthora infestans* compared to the wild-type (Kühnlenz et al., 2015).

All this assumed, it should also be considered that the phenylpropanoid pathway is a starting point for the production of a specialized number of metabolites such as lignins, sinapates, and flavonoids; and that some investigations have shown the existence of a possible crosstalk between glucosinolate and phenylpropanoid metabolic pathways (Hemm et al., 2003; Kliebenstein et al., 2005; Kim et al., 2015). For example, the incorporation of the sinapoyl moiety might occur through a sinapoyl-CoA co-substrate leading to the biosynthesis of sinapoylated glucosinolates (Sønderby et al., 2010). Besides, Kim et al. (2015) showed that *ref5-1* mutants (*A. thaliana reduced epidermal fluorescence5*), which are defective in the accumulation of soluble phenylpropanoids, had a missense mutation in CYP83B1, a key enzyme in indole glucosinolate biosynthesis.

Consequently, the lack of PCS1 (as occurs in the *cad1-3* mutant) might alter both the glucosinolate and, the phenylpropanoid pathway, the latter having potential repercussions on metabolism of monolignols, the degree of lignification, and the biosynthesis of other phenolic compounds, i.e., flavonoids. In fact, Cd treatment upregulated the transcript levels of genes encoding monolignol biosynthetic enzymes in both roots and shoots (Herbette et al., 2006), suggesting subsequent increases in lignin biosynthesis. Interestingly, some flavonoids can act as negative regulators of auxin movement *in vivo* (Murphy et al., 2000; Brown et al., 2001; Peer et al., 2001; Buer and Muday, 2004; Buer et al., 2013; Kuhn et al., 2016), and therefore variations in their levels might affect auxin content and distribution *in planta*.

To revisit the role of PCS1 in plants and to elucidate its function in the secondary metabolism, we compared here the metabolome of *A. thaliana* wild-type and PCS1-deficient *cad1-3* mutant. Indeed, we reported data showing that – in addition to the glucosinolate pathway – AtPCS1 plays a role in regulating the phenylpropanoid pathway. In the glucosinolate pathway, AtPCS1 influences callose deposition, with a consequent increased resistance to high Cd concentrations and to the hemibiotrophic bacterial pathogen *Pseudomonas syringae*. In the phenylpropanoid pathway, AtPCS1 modulates the levels of hydroxycinnamic acids, lignin and flavonols, with consequent variations in auxin content, both in shoots and in roots.

## Materials and Methods

### Plant Material and Growth Conditions

*Arabidopsis thaliana* (L.) Heynh. lines employed in this work were all in Col-0 background and obtained from seeds of wild-type, *cad1-3* mutant (Howden et al., 1995), *pen2-1* mutant (Lipka et al., 2005) and *pad4* mutant (Glazebrook et al., 1997).

For metabolomics and auxin identification and quantification, *A. thaliana* wild-type and *cad1-3* plants (200 plants for each line) were axenically grown for 20 days in Petri dishes supplied with solid Gamborg’s B-5 Basal Salt Mixture medium (Sigma–Aldrich, St. Louis, MO, United States), supplemented with Gamborg’s vitamin solution (Sigma–Aldrich), sucrose (30 g L^-1^) and 7 g L^-1^ agar. The pH of the final solution before adding agar was adjusted to 5.8. The plant material was kept at 22 ± 1°C under 14 h light, at a photosynthetic photon flux density of 120 μmol m^-2^ s^-1^, with 60% relative humidity. On the 20th day, half of the plants (both wild-type and *cad1-3*) were supplied with a cadmium chloride (CdCl_2_) solution – by overlying the solid B5 medium – to reach a final concentration of 36 μM Cd, whereas the other half was provided with the same amount of bidistilled ultrapure water (0 μM Cd), with a resistivity of 18.2 MΩ cm. Control and Cd-treated plants were kept under these conditions for additional 24 h. Twenty plants for each line and condition were designated as a sample, and five biological replicates (*n* = 5) were used for each HPLC-ESI-MS analysis. Shoots and roots were separated, carefully washed with bidistilled water, frozen in liquid nitrogen, and stored at -80°C, prior to further procedures.

For callose staining (detailed in section “Callose Staining”), wild-type, *cad1-3* and *pen2-1* seedlings were grown for 10 days in B5 medium, set up as described above. For analysis of Cd sensitivity (root length and plant growth), wild-type, *cad1-3* and *pen2-1* plants were grown for 14 days in B5 medium, containing 0 (control), 25, 50, and 75 μM CdCl_2_. For pathogen resistance assays (detailed in section “Bacterial Growth Assays”), wild-type, *cad1-3* and *pad4* plants were grown in Metro-Mix soil (Sun Gro, Agawam, MA, United States) for 28 days. For stem stretch-resistance assays and lignin staining (detailed in section “Stretch Assays and Lignin Staining”), wild-type and *cad1-3* plants were grown in Metro-Mix soil (Sun Gro, Agawam, MA, United States) for 2 months.

### Metabolite Extraction and HPLC-ESI-MS Analyses

Frozen shoots and roots of wild-type and *cad1-3* plant (150 mg each) were powdered in liquid nitrogen and the metabolites extracted on ice by means of four volumes of cold methanol (w/v), followed by vigorous mixing with a “vortex” type mixer and sonication at 40 kHz for 15 min in an ultrasonic bath (Falc Instruments, Treviglio, Italy). Samples were then centrifuged at 16,000 × *g* for 10 min at 4°C, and supernatants were filtered through minisart RC4 (0.2 μm) pore filters. The methanol phases were diluted with HPLC-MS grade water (1/3, v/v) (Sigma–Aldrich), and analyzed by reverse phase HPLC-ESI-MS. Fragmentation experiments were performed in both positive and negative ion modes, by using a Beckman Coulter Gold 127 HPLC system (Beckman Coulter, Fullerton, CA, United States) equipped with a C18 guard column (7.5 mm × 2.1 mm) and an analytical Alltima RP C18 column (150 mm × 2.1 mm, particle size 3 μm) (Alltech Associates Inc., Deerfield, IL, United States). The solvents used for HPLC-ESI-MS analysis were 0.5% (v/v) formic acid, 5% (v/v) acetonitrile in water (solvent A), and 100% acetonitrile (solvent B). A solvent gradient was established from 0 to 10% B in 5 min, from 10 to 20% B in 10 min, from 20 to 25% B in 5 min, and from 25 to 70% B in 15 min. The injection volume was equal to 5.0 μL, and the flow rate was 200 μL min^-1^. The HPLC system was coupled on-line with a Bruker ion trap mass spectrometer Esquire 6000, equipped with an electrospray ionization source. MS data were collected using the Bruker Daltonics Esquire Control 5.2 software, and processed using the Bruker Daltonics Esquire 5.2-Data Analysis 3.2 software (Bruker Daltonik GmbH, Bremen, Germany). The alternate mass spectra were recorded in the range 50–3000 m/z (full scan mode, 13.000 m/z s^-1^). For the fragmentation pattern analysis, MS/MS and MS3 spectra were recorded in negative and positive mode in the range 50–3000 m/z, with the fragmentation amplitude set at 1 V. Nitrogen was used as the nebulizing gas (0.34 MPa, 350°C) and drying gas (10 L min^-1^), and helium as the collision gas.

The metabolites were identified through a comparison of m/z, retention time and fragmentation pattern (**Supplementary Table S1**) with an in house library of authentic standard and through a comparison of m/z and fragmentation pattern with the data available in the MassBank public database^1^ and in the literature. Chromatogram data extraction and alignment were carried out using MZmine software^2^. The relative quantification (i.e., comparison between samples) was based on the area of each of the signals extracted from the chromatograms and expressed as arbitrary units (a.u.) intensity.

Once all metabolic profiles from wild-type and *cad1-3* plants were obtained, data were processed using the statistical SIMCA-P+12 software (Umetrix AB, Umeå, Sweden). Two main methods were used: the Principal Component Analysis (PCA), and the Orthogonal Projection to Latent Structures Discriminant Analysis (O2PLSDA). In both cases the Pareto scaling was used. In detail, this O2PLSDA-S loading plot analysis (**Supplementary Figure S1**) correlates the metabolites with the operator defined group of samples (classes, in this case wild-type and *cad1-3* mutant) and highlights the differences in terms of metabolite accumulation among them. Models were cross-validated using a permutation test (200 permutations). The average value of the two technical replicates of each sample was calculated for each metabolite. Statistics of the metabolome analysis was further completed as detailed in Section “Statistics.”

### Callose Staining

Callose staining was performed in cotyledons, as described in Clay et al. (2009). Briefly, 10-day-old seedlings of wild-type, *cad1-3, pen2-1* (grown as described in section “Plant Material and Growth Conditions”) were incubated for 24 h in bidistilled ultrapure water, or 1 μM Flg22 (a 22-amino acid sequence-long N-terminal part of flagellin sufficient to activate plant defense mechanisms), or 25 μM CdCl_2_. Seedlings were then fixed in a solution containing ethanol and glacial acetic acid (3:1 ratio, respectively) using short-term vacuum infiltration, and placed on a shaking platform for about 4 h (until cotyledons appeared slightly translucent), with several changes of fixing solution. Seedlings were subsequently rehydrated in 70% ethanol for at least 2 h, then in 50% ethanol, washed twice in deionized water, and left overnight in water on a shaking platform. Afterward, seedlings were made transparent in 10% NaOH, which was introduced by using vacuum infiltration for several minutes, then washed-free from NaOH with deionized water, incubated for 5 h in a 150 mM K_2_HPO_4_ solution (pH 9.5) containing 0.01% aniline blue and fixed on slides with 50% glycerol.

Callose-mediated fluorescence was visualized using a DAPI filter set (excitation filter 390 nm; dichroic mirror 420 nm; emission filter 460 nm) of an Axio Imager M2 microscope equipped with the motorized Z-drive (Zeiss, Oberkochen, Germany). Images were collected with AxioCam MR Camera and processed using the Adobe Photoshop software package, version 12.0. The ImageJ software (SciJava software ecosystem, *open source*) was used to quantify the intensity and the number of callose deposits.

### Bacterial Growth Assays

*Pseudomonas syringae* DC3000 cells were grown at 30°C in lysogeny broth (LB) medium containing rifampicin at a concentration of 50 μg mL^-1^. Prior to infiltration, bacteria were suspended in sterile 10 mM MgCl_2_ and bacterial cell density (OD_600_) was measured using a Jenway 6320D spectrophotometer (Bibby Scientific Limited, Staffordshire, United Kingdom). *P. syringae* DC3000 was infiltrated at a concentration of 3⋅10^5^ bacterial colony forming units (CFU) mL^-1^ in leaves of 28 day-old wild-type, *cad1-3*, and *pad4* plants, all grown in Metro-Mix soil. To determine the bacterial propagation on leaves, the internal bacterial population was measured in 12 biological replicates (*n* = 12) at time 0 and 48 h after infiltration, and each replicate represented a pool of three leaves from the same infiltrated plant. Specifically, inoculated leaves were collected by cutting the leaf with a punch (diameter 6 mm) and all samples were transferred to a 96-well plate containing 1 mL of 10 mM MgCl_2_ buffer and stainless steel beads. After homogenization in a shaker chamber, the extracts were fivefold serially diluted (1:10) and plated on petri dishes with LB medium containing rifampicin. The plates were then incubated for 2 days at 30°C and CFU was counted using the diluted samples. Bacterial populations were evaluated using three independent experiments.

### Stretch Assays and Lignin Staining

Stems of wild-type and *cad1-3* 2-month-old plants were sectioned at the level of basal, middle and upper zones. Afterward, the pressure (MPa) required to break them was measured with the Instron instrument (Norwood, MA, United States), mainly following Xiao et al. (2017). Detection of lignin was performed on microtome-cut (Swift Microtome MA501, London, United Kingdom) sections of stems (basal zone) collected from the above material, stained with 1% phloroglucinol (w/v) in 12% HCl for 5 min, according to Weng et al. (2010). Lignin was visualized using a light Axio Imager M2 microscope equipped with the motorized Z-drive (Zeiss). Images were collected with AxioCam MR camera and processed using the Adobe Photoshop software package, version 12.0. Morphometric analysis of lignified areas of stems was performed with the ImageJ software, by manually cropping the phloroglucinol-HCl stained areas.

### Auxin Identification and Quantification

For extraction of IAA, IBA, ICA, and MeIAA, 100 mg of root and shoot tissues were collected from 21-day-old wild-type and *cad1-3* plants, grown without Cd. Plant tissues were homogenized on ice in a mortar with 1 mL of 2-propanol/H_2_O/HCl 37% (2:1:0.002, v/v/v). One mL of dichloromethane was added to each sample, mixed and centrifuged at 13,000 × *g* for 5 min at 4°C. The lower phase was removed (750 μL), concentrated using an evaporator with nitrogen flow, and then re-dissolved in 15 μL of methanol. Auxins were identified by a LC-MS/MS Shimadzu LCMS-8045 triple quadrupole system (Shimadzu Co., Kyoto, Japan), according to Sofo et al. (2011). Mass spectrometry data for ICA, IAA, and IBA were acquired in the negative ionization mode, whereas for MeIAA in the positive ionization mode (**Supplementary Table S2**). Pure standards of the above hormones (Duchefa Biochemie B.V., Haarlem, Netherlands) were used for quantification. All auxins were determined by calculating the correction factor for each authentic hormone in comparison with its corresponding internal standard, namely [^2^H_5_] IAA, [^2^H_9_] IBA, [^2^H_5_] ICA, and [^2^H_5_] IAA methyl ester (OlChemIm Ltd., Olomouc, Czechia).

### Statistics

**FIGURE 1:**
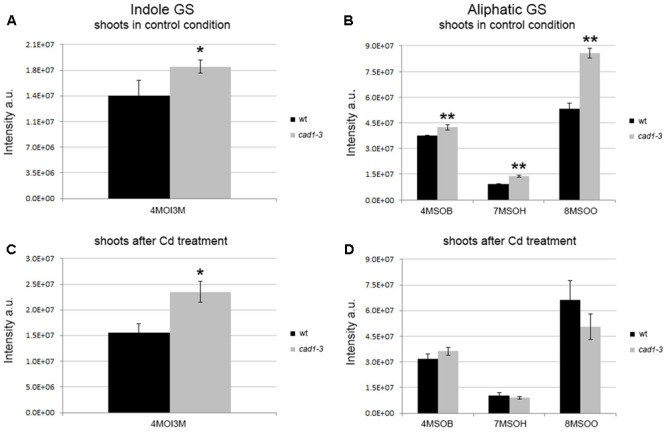
Levels of indole and aliphatic glucosinolates in shoots from wild-type (wt) and *cad1-3* plants, grown under control conditions **(A,B)** or exposed to 36 μM CdCl_2_ for 24 h **(C,D)**. 4MOI3M, 4-methoxy-indol-3-ylmethylglucosinolate; 4MSOB, 4-methylsulfinylbutylglucosinolate; 7MSOH, 7-methylsulfinylheptylglucosinolate; 8MSOO, 8-methylsulfinyloctylglucosinolate. The glucosinolate relative content is reported as ion counts (arbitrary units, a.u.). *n* = 5, error bars indicate SE. Statistically significant differences are calculated for each metabolite, comparing wt with *cad1-3* (in the absence or presence of Cd), by means of *t*-student test, 2-tailed distribution. ^∗^*p* ≤ 0.05, ^∗∗^*p* ≤ 0.01.

**FIGURE 2:**
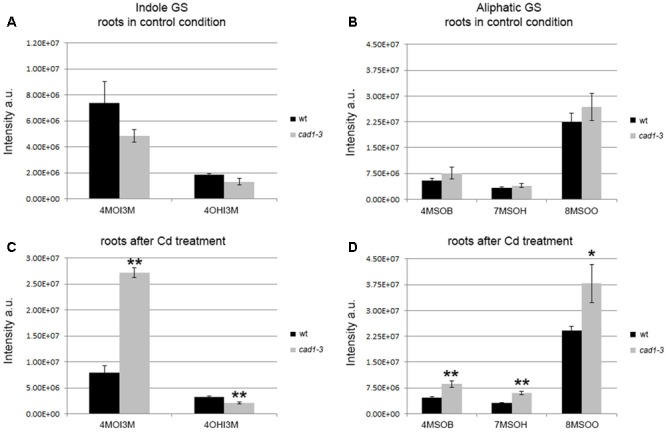
Levels of indole and aliphatic glucosinolates in roots from wt and *cad1-3* plants, grown under control conditions **(A,B)** or exposed to 36 μM CdCl_2_ for 24 h **(C,D)**. 4OHI3M, 4-hydroxy-indolyl-3-methylglucosinolate; other glucosinolate abbreviations and statistics as in **Figure 1**.

**FIGURE 3:**
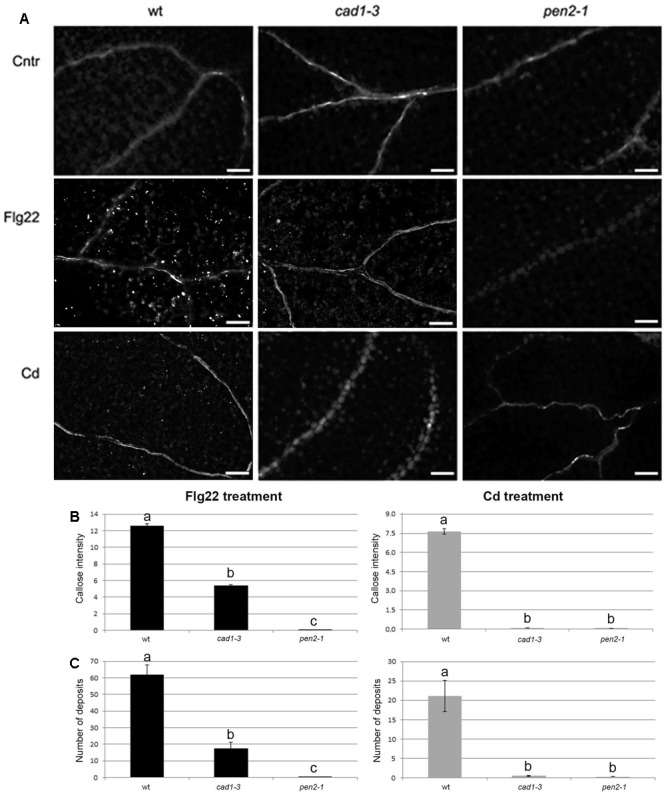
Callose staining in cotyledons of 10-day-old wt, *cad1-3* and *pen2-1* plants treated for 24 h with double-distilled water (Cntr), or 1 μM Flg22, or 25 μM CdCl_2_. **(A)** Representative images of aniline blue-stained callose fluorescence (bright spots); *n* > 6, scale bars = 100 μm. **(B)** Callose fluorescence intensity and **(C)** number of callose deposits, in the three lines and conditions. In **(A,B)**, the values represent the average of *n* > 6, error bars indicate SE. Statistical analysis was performed by one-way ANOVA followed by the Bonferroni’s *post hoc* test. Different letters indicate statistically significant differences at *p* ≤ 0.05.

**FIGURE 4:**
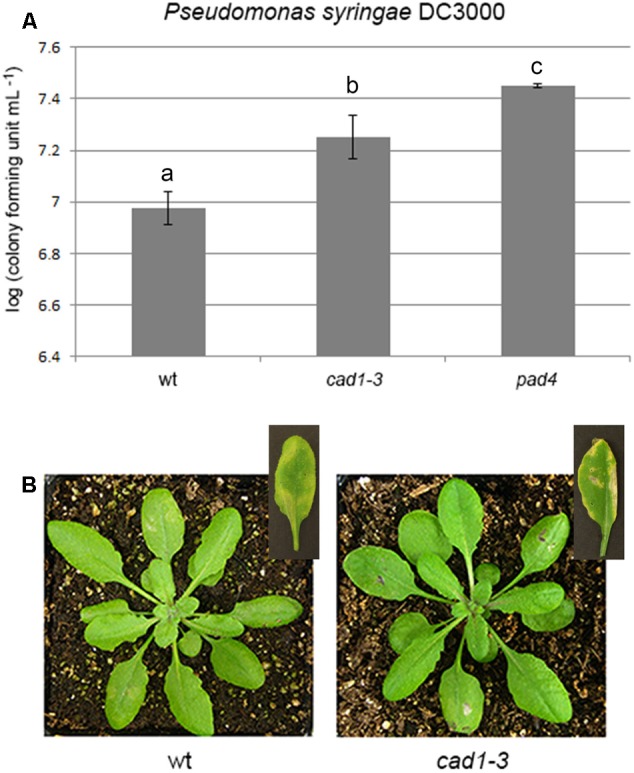
Bacterial growth **(A)** and induced disease symptoms **(B)** of *Pseudomonas syringae* DC3000 infiltrated for 48 h, at a concentration of 3⋅10^5^ bacterial colony forming units mL^–1^, in leaves of 28-day-old wt, *cad1-3*, or *pad4* plants. In the insets of **(B)**, representative leaves 7 days after the bacterial infiltration are shown. *n* = 12, error bars indicate SE. Statistical analysis was performed by one-way ANOVA followed by the Bonferroni’s *post hoc* test. Different letters indicate statistically significant differences at *p* ≤ 0.05.

**FIGURE 5:**
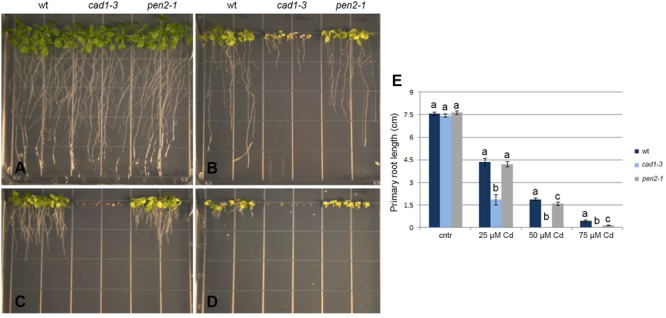
Phenotype of wt, *cad1-3* and *pen2-1* two-week-old plants grown with 0 (cntr) **(A)**, 25 **(B)**, 50 **(C)**, and 75 μM **(D)** CdCl_2_ for 14 days. **(E)** Primary root length of the same plants. *n* = 14, error bars indicate SE. Statistical analysis was performed for each Cd concentration by one-way ANOVA followed by the Bonferroni’s *post hoc* test. Different letters indicate statistically significant differences at *p* ≤ 0.05.

**FIGURE 6:**
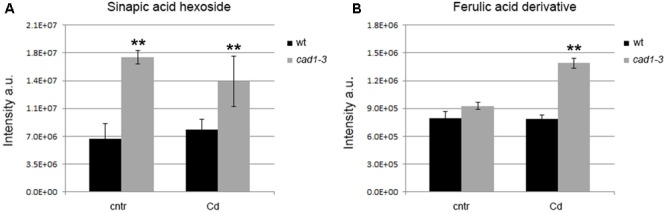
Levels of sinapic acid hexoside in shoots **(A)** and ferulic acid derivative in roots **(B)** of wt and *cad1-3* plants, grown under control conditions (cntr) or exposed to 36 μM of CdCl_2_ for 24 h. The metabolite relative content is reported as ion counts (arbitrary units, a.u.). *n* = 5, error bars indicate SE; statistics as in **Figure 1**.

**FIGURE 7:**
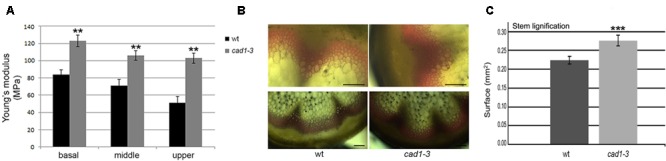
**(A)** Sections from basal, middle and upper zones of wt and *cad1-3* stems subjected to stretch. Pressure (MPa) required by the Instron instrument to break the stems is shown. *n* = 6, error bars indicate SE; statistics as in **Figure 1**. **(B)** Stem sections from the basal zone stained with phloroglucinol. The reddish color visualizes lignin accumulation. Representative results of three independent experiments are shown. Scale bars = 100 μm. **(C)** Morphometric analysis on lignified area (mm^2^) from stem sections (basal zone) performed with ImageJ software. *n* = 6, error bars indicate SE; statistics as in **Figure 1**. ^∗∗∗^*p* ≤ 0.001.

**FIGURE 8:**
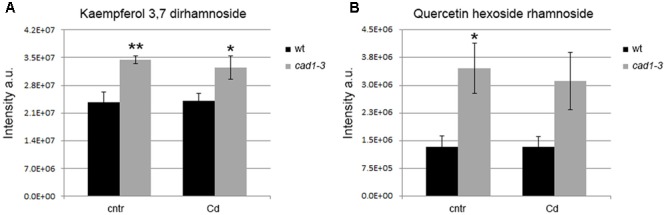
Levels of kaempferol 3,7 dirhamnoside **(A)** and quercetin hexoside rhamnoside **(B)** in shoots of wt and *cad1-3* plants, grown under control conditions (cntr) or exposed to 36 μM of CdCl_2_ for 24 h. The metabolite relative content is reported as ion counts (arbitrary units, a.u.). *n* = 5, error bars indicate SE; statistics as in **Figure 1**.

**FIGURE 9:**
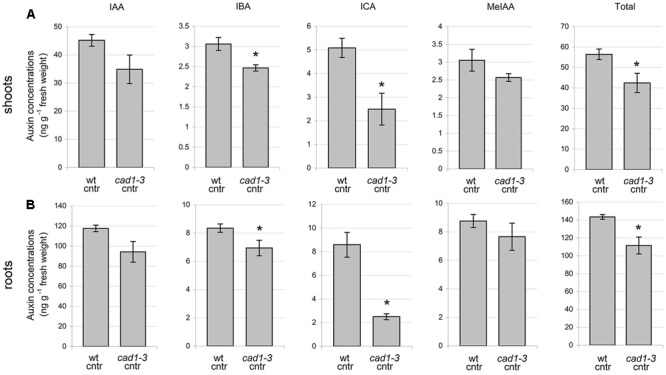
Concentration of auxins in shoots **(A)** and roots **(B)** of wt and *cad1-3* control (cntr) plants. IAA, indole-3-acetic acid; IBA, indole-3-butyric acid; ICA, indole-3-carboxylic acid; MeIAA, indole-3-acetic acid methyl ester. *n* = 3, error bars indicate SE; statistically significant differences assessed by the non-parametric Mann–Whitney *U*-test; ^∗^*p* ≤ 0.05.

For **Figures 1, 2, 6, 7A–C, 8**, statistically significant differences were calculated for each metabolite (Young’s modulus or stem lignification for **Figure 8**), comparing the wild-type with the *cad1-3* (in the absence or presence of Cd) by the *t*-student test, 2-tailed distribution. For the multiple comparisons of **Figures 3–5**, the one-way ANOVA was performed, followed by the Bonferroni *post hoc* test. For the non-normally-distributed data of **Figure 9**, the non-parametric Mann–Whitney *U*-test was applied. All statistical analyses were performed by the SigmaStat version 13.0 software (Systat Software Inc., Chicago, IL, United States).

## Results

### AtPCS1 Influences the Relative Levels of Glucosinolate Both in Shoots and Roots

The untargeted metabolomics revealed 100 m/z features in the shoots and 217 m/z in the roots. These m/z features were detectable in almost all samples, with the sole exception of one feature in the shoots and two features in the roots which were undetectable in wild-type samples without Cd. As a preliminary analysis, the average relative levels of each m/z feature in wild-type and *cad1-3* samples was examined in the presence or absence of Cd. The metabolites that showed at least the doubling of the relative levels from wild-type to *cad1-3* (or vice versa) (i.e., those metabolites that showed the highest variation between samples) were considered for further investigation. This analysis showed 18 metabolites in the shoots (8 in the absence and 13 in the presence of Cd) and 35 metabolites in the roots (16 in the absence and 21 the in presence of Cd) that at least doubled their relative levels from wild-type to *cad1-3* or vice versa.

The distinct clusters of metabolites from wild-type and *cad1-3* plants (shoots and roots) along the first two principal components in O2PLS-DA analysis (**Supplementary Figure S1**) show the effect of AtPCS1 on the secondary metabolism. The identified metabolite list is reported in **Supplementary Table S1**.

In general, both indole and aliphatic glucosinolates were produced at higher relative levels in shoots from *cad1-3* plants, compared to wild-type (**Figures 1A,B**). The exposure to Cd did not substantially change this scenario, except for a further stimulated production of the indole glucosinolate 4MOI3M compared with the respective control (**Figure 1C**). Moreover, the relative levels of aliphatic glucosinolates with short carbon chain length, that is 4MSOB, and with long carbon side chains, namely 7MSOH and 8MSOO, increased significantly in shoots of *cad1-3* compared with the wild-type, both grown under control conditions (**Figure 1B**). By contrast, no differences in aliphatic glucosinolate relative levels were observed in shoots from Cd-treated wild-type and *cad1-3* plants (**Figure 1D**).

The overall picture of indole and aliphatic glucosinolates appeared to be more articulated in roots than in shoots, and overall their relative levels appeared to be more influenced by Cd treatment (**Figure 2**). Specifically, the 4OHI3M decreased in *cad1-3*, whereas the 4MOI3M increased considerably, both only after Cd treatment (**Figure 2C**). Concerning aliphatic glucosinolates, the relative levels of 4MSOB, 7MSOH, and 8MSOO were higher in roots of *cad1-3* vs. wild-type plants only after Cd treatment (**Figure 2D**), whereas no differences were observed under control conditions (**Figure 2B**).

### AtPCS1 Is Essential for Callose Deposition under Cd Stress and Is Involved in Protection against the Bacterial Pathogen *P. syringae*

In wild-type seedlings, callose deposition was triggered by both Flg22 and Cd exposure, with a fluorescence intensity and a number of callose deposits, respectively, 1.7- and 2.9-fold higher in the Flg22-triggered seedlings compared with the Cd-treated ones (**Figures 3B,C**). By contrast, *cad1-3* and *pen2-1* mutants were both defective in callose deposition compared with the wild-type, after both treatments (**Figure 3**). However, whilst the *pen2-1* mutant showed nearly no callose fluorescence intensity and deposits after Flg22 and Cd exposure (**Figures 3B,C**), the *cad1-3* displayed a reduced degree of callose intensity (less than 47% of the wild-type one) and a few callose deposits (less than 30% of wild-type ones) in the presence of Flg22, but not with Cd (**Figures 3B,C**).

As a possible consequence of the impaired callose deposition, *cad1-3* plants were more sensitive to the bacterial pathogen *P. syringae* DC3000 (*Ps*DC3000) than wild-type, regardless of whether the sensitivity was measured as the number of colonies formed by *Ps*DC3000 (**Figure 4A**) or as the extension/severity of lesions on the leaf surface (**Figure 4B**). The pathogen-sensitive *pad4* mutant (Xing and Chen, 2006), used as a control, was more colonized by the pathogen than wild-type and *cad1-3* (**Figure 4A**).

To test the potential contribution of callose against Cd toxicity, plant growth, total size of the root system and primary root length were measured in wild-type, *cad1-3* and *pen2-1* lines, grown for 14 days in B5 medium in the presence of 0, 25, 50, or 75 μM Cd. The growth of all lines was indistinguishable under control conditions (**Figure 5A**), whereas increasing Cd concentrations progressively inhibited the overall growth and the (primary) root length (**Figures 5A–E**). Marked leaf chlorosis and necrosis were detected in a Cd concentration-dependent manner (**Figures 5B–D**). The *cad1-3* plants were extremely sensitive to Cd, regardless of the concentration used (**Figure 5E**), whereas the *pen2-1* were much less sensitive to the metal than *cad1-3*, but more sensitive than wild-type at the highest Cd concentrations (50 and 75 μM; **Figure 5E**).

### Lack of PCS1 Alters the Phenylpropanoid Pathway with Consequences on the Degree of Lignification, Flavonol Content and Auxin Level

Another major metabolic change in the *cad1-*3 mutant as compared to the wild-type plants occurred in the phenylpropanoid pathway. In particular, sinapic acid hexoside was present at higher relative levels in shoots of *cad1-3* plants compared with wild-type, both grown in control conditions and in the presence of Cd (**Figure 6A**). Likewise, a ferulic acid derivative was accumulated at higher relative levels in *cad1-3* roots vs. wild-type as well, but only after Cd treatment (**Figure 6B**).

Since these metabolites are involved in lignin biosynthesis (Lim et al., 2001; Boerjan et al., 2003), we hypothesized that the observed changes would affect the cell wall properties of the *cad1-3* plants. To test this, we examined whether wild-type and *cad1-3* plants differed in stem stretch resistance, thus reflecting their respective cell wall rigidity. Accordingly, stems of 2-month-old plants were stretched in the basal, middle (at the second internode) and upper (at the third–fourth internode) stem zones. An increased resistance to the applied stretch in stems of *cad1-3* vs. wild-type was detected, in all zones subjected to the test (**Figure 7A**). We then verified whether differences in cell wall properties of the stems of *cad1-3* vs. wild-type were due to altered lignification; lignin accumulation in the two plant lines was compared using phloroglucinol staining. We found that the stained area of the *cad1-3* appeared thicker and the color was more intense compared to wild-type, in particular at the stem’s basal zone (**Figure 7B**). The morphometric analysis of the lignified area confirmed that the increased stretch resistance of the basal parts of the stems in the *cad1-3* mutant was due to a significant increase in the degree of lignification (**Figure 7C**).

In addition to precursors of lignin biosynthesis, the accumulation of other compounds produced by the phenylpropanoid pathway, namely flavonols, differed between wild-type and *cad1-3* lines shoots. Specifically, a higher relative level of kaempferol 3,7 dirhamnoside was found in *cad1-3* shoots compared to wild-type, both under control conditions and Cd exposure (**Figure 8A**). Likewise, the concentration of quercetin hexoside rhamnoside under control conditions was higher in shoots of *cad1-3* vs. wild-type. This was observed, at least as an upward trend, also after Cd treatment, with a significance level of *p* ≤ 0.06 (**Figure 8B**). By contrast, no differences in flavonol content between wild-type and *cad1-3* were detected in roots.

To analyze whether the higher flavonol levels found in the *cad1-3* mutant might affect auxin content, the concentrations of IAA, IBA, ICA, and MeIAA were measured in *cad1-3* and wild-type plants, both grown under control condition. The total auxin concentrations, and in particular the IBA and ICA ones, were significantly lower in roots and shoots of the *cad1-3* mutant compared with the wild-type (**Figure 9**).

## Discussion

Overall, our results are consistent with the role of the AtPCS enzyme in the accumulation of indole and aliphatic glucosinolates (Schlaeppi et al., 2008). To this end, in agreement with Clay et al. (2009), we found an increased level of 4MOI3M in shoots of *cad1-3* plants grown under control conditions. 4MOI3M has deterrent properties, and its breakdown products contribute to the defense against pathogens and herbivores (Halkier and Gershenzon, 2006; Kim and Jander, 2007; Bednarek et al., 2009). Our results revealed an increase in 4MOI3M in *cad1-3* plants also after Cd exposure, by a factor of about 1.5 and 4 in shoots and roots, respectively. These data thus highlight that the 4MOI3M glucosinolate plays a key role in the activation of common response pathways toward Cd toxicity and biotic stress. We found an accumulation of 4MSOB, 7MSOH, and 8MSOO in shoots of *cad1-3* plants grown in control conditions, as well as in roots of this mutant after Cd treatment, presumably as a consequence of the 4MOI3M relatively high accumulation. Likewise, according to Kim and Jander (2007), the accumulation of 4MOI3M after aphid infestation of *A. thaliana* leaves significantly stimulated the synthesis of some aliphatic glucosinolates.

To test if the alteration of the glucosinolate pathway in *cad1-3* affects plant response to bacterial pathogens, other than to the oomycete *Phytophthora infestans* (Kühnlenz et al., 2015), we compared the sensitivity of wild-type and *cad1-3* plants to the hemibiotrophic bacterium *P. syringae* DC3000. Callose is one of most important mechanism of defense responses to pathogens, and the *cad1-3* mutant shows defects in callose deposition after Flg22 treatment. As expected, our *cad1-3* plants were more susceptible to *Ps*DC3000 than wild-type. A similar trend for the same pathogen was found for the *pen2-1* mutant which accumulates 4MOI3M and is impaired in callose deposition. We used as a control the *pad4* mutant, which is more sensitive to pathogen as well (Xing and Chen, 2006). Therefore, the catabolic products of 4MOI3M are components of the defense response toward biotic stress in *A. thaliana.* We found that this response mechanism involves AtPCS1.

The synthesis of callose is also activated under exposure to toxic heavy metals (Ueki and Citovsky, 2002, 2005), but callose production after Cd treatment has never been experimentally validated. We noticed that Cd exposure induces callose deposition only in wild-type plants, but this event was shown to be not as prominent as following Flg22 treatment. We also found that Cd treatment did not stimulate callose deposition in *cad1-3* and *pen2-1* mutants. The lack of stimulation could be due to the remarkable accumulation of 4MOI3M in *cad1-3* plants, also as a response to metal stress. These results highlight that AtPCS1 protects plants against heavy metal toxicity not only by producing PCs, but also by contributing to callose deposition. The proposed pathway may involve the conjugation of isothiocyanates (which are the products of PEN2-catalyzed glucosinolate hydrolysis) with GSH, and the subsequent glycine cleavage by AtPCS1 (Bednarek et al., 2009; Kühnlenz et al., 2015). In addition, our data suggest the importance of callose deposition in protecting plants particularly against high concentrations of Cd, since the callose-defective mutant *pen2-1* showed an increased sensitivity to 50 and 75 μM Cd, compared with wild-type plants.

Interestingly, our metabolomic study also showed an upregulation of the phenylpropanoid pathway in *cad1-3* plants. Specifically, accumulation of sinapic acid hexoside was found in shoots of *cad1-3* plants, both under control conditions and exposed to Cd. Sinapic acid is involved in lignin biosynthesis (Lim et al., 2001). In fact, in line with our findings, exposure to Cd upregulated the transcript levels of genes encoding proteins involved in monolignol synthesis (Herbette et al., 2006), suggesting a possible consequent increase in lignin biosynthesis as well. In general, an increased lignification can be interpreted as an avoidance mechanism aimed at reducing the metal entry inside the cells, the inner tissues, the xylem flow, etc. The increase in the cell wall thickness can in fact lead to a substantial extension of the surfaces potentially useful for the immobilization of toxic metals (Van Belleghem et al., 2007; Sanità di Toppi et al., 2012). Since *cad1-3* plants are not able to produce PCs and are impaired in callose deposition, the increased levels sinapic acid hexoside could result in an augmented lignin biosynthesis. This evidence was supported by the increased resistance of the *cad1-3* stems to stretch, indicative of rigidity increments of cell walls, and also by the increase in lignin staining. Thus, an augmented lignification can represent a further mechanism for counteracting Cd toxicity in *cad1-3* plants, which lack a major PC-dependent, and in part an auxiliary callose-dependent, heavy metal protective systems.

In our experiments, besides an increased level of sinapic acid hexoside in the shoots, Cd stress also raised the levels of a ferulic acid derivative in the roots of *cad1-3* plants vs. wild-type. Intriguingly, such increase could be one explanation for the strong decrease in primary root length observed in the former. In fact, the formation of diferulic linkages typically occurs at various stages during cell wall formation (Passardi et al., 2004). Changes in the accumulation of cell wall-bound phenolics, such as ferulic acid derivatives, are typically reported under abiotic stress conditions (i.e., drought and salt stress) and are involved in the progressive inhibition of root growth (Fan et al., 2006; Neves et al., 2010). As a consequence, the reduction in root growth possibly mediated by the ferulic acid derivative accumulation may facilitate the root response to toxic metal exposure.

Although glucosinolates and phenylpropanoids are synthesized through different biosynthetic pathways and have distinct functions, some evidence indicates a link between these two pathways (Hemm et al., 2003; Kim et al., 2015). In fact, genetic and biochemical investigations suggested that the crosstalk between the indole glucosinolate pathway and the early steps of phenylpropanoid biosynthesis could be regulated by the levels of IAOx or by a subsequent metabolite (Kim et al., 2015). On this basis, we may surmise that also in our experiments the increased content in sinapic acid hexoside in the shoots might be related to a possible decrease in the IAOx content (not measured). In addition, it may have taken place a carbon re-routing toward phenylpropanoid biosynthesis caused by lack of PCS, which might have rendered plants in a pseudo-state of stress responsiveness even in the absence of Cd.

We also found increased levels of other metabolites from the phenylpropanoid pathway, particularly some flavonols. In fact, two glycosylated forms of kaempferol and quercetin were detected at higher relative levels in *cad1-3* shoots, compared with wild-type ones. These flavonols regulate auxin retention and transport *in vivo* (Murphy et al., 2000; Brown et al., 2001; Peer et al., 2001; Buer and Muday, 2004; Buer et al., 2013). Experiments using an allelic series of *tt4* mutants, with defects in the gene encoding chalcone synthase and characterized by the absence of flavonoids, have revealed that these phenolic compounds likely alter auxin transport in *A. thaliana* (Brown et al., 2001). Indeed, flavonols act as regulators of cellular auxin efflux and consequent auxin polar transport (Murphy et al., 2000; Buer and Muday, 2004; Peer and Murphy, 2007; Rusak et al., 2010).

To analyze whether the higher flavonol levels found in *cad1-3* plants affect auxin levels in shoots and roots, we measured the concentrations of auxins (IAA, IBA, ICA, and MeIAA) in wild-type and *cad1-3* plants, grown under control conditions. Interestingly, the *cad1-3* mutant accumulated lower levels of total auxins in shoots and roots compared with wild-type plants. The abundance of kaempferol and quercetin derivatives found in *cad1-3* shoots is in fact well correlated with the lower auxin level of this mutant, both in shoots and in roots. In agreement with our findings, kaempferol 3-*O*-rhamnoside-7-*O*-rhamnoside has been recently identified as an endogenous polar auxin transport inhibitor in *A. thaliana* shoots in a dose-dependent manner (Yin et al., 2014). Also, the formation of kaempferol 3-*O*-rhamnoside-7-*O*-rhamnoside is subordinated to a specific 3-*O*-rhamnosyltransferase, which is relatively abundant at the *A. thaliana* shoot apex, but almost absent in the roots (Jones et al., 2003). In addition, Kuhn et al. (2016) indicated that flavonols, and in particular 7-rhamnosylated ones, influence auxin transport and turnover, thus modifying the overall auxin levels *in planta*. Indeed, flavonoids can also accumulate in target-tissues far from the synthesis site, indicating that intermediates in their pathway can move long distances (Buer et al., 2007, 2008). Flavonoids have also been demonstrated to effectively modulate auxin signaling, not only by regulating auxin gradient (Grunewald et al., 2012) and flux (Michniewicz et al., 2007), but by buffering the formation of reactive oxygen species (ROS) (Peer et al., 2013) and ROS-dependent auxin oxidation (Peer et al., 2013). In our experiments, the flavonols might have regulated the auxin content at different levels, both modulating intra- and cell-to-cell auxin movements (Adamowski and Friml, 2015) and reducing auxin oxidation (Peer et al., 2013).

Not least, some intermediate steps in indole glucosinolate biosynthesis are shared not only with the biosynthesis of camalexin, which was found to be less accumulated in *cad1-3* plants vs. wild-type after pathogen infection (Su et al., 2011), but also with ICA biosynthesis (Böttcher et al., 2014), which plays also an important role in plant–pathogen defense response (Böttcher et al., 2009, 2014). The significantly lower concentration of ICA in *cad1-3* plants compared with wild-type may be directly related to lack of AtPCS1, because ICA is synthesized in the glucosinolate pathway through *A. thaliana* aldehyde oxidase1 (AAO1) and Cytochrome P450 (CYP)71B6, starting from IAN (Böttcher et al., 2014). Since AAO1 and CYP71B6 are transcriptionally coexpressed with camalexin biosynthetic genes, the role of ICA has been recently revised, not simply as an IAA catabolite, but also as a metabolite involved in induced chemical defense against biotic agents (Böttcher et al., 2014).

It has been shown that camalexin is derived from IAOx, which is synthesized from tryptophane by the cytochrome P450 enzymes CYP79B2 and CYP79B3 (Glawischnig et al., 2004). IAOx is also an intermediate in the biosynthesis of indole glucosinolates and a precursor for IAA. This makes IAOx a possible key point in the branching of different metabolic pathways. Other evidence has shown that IAOx is a precursor of IAA, and because of defects in CYP83B1 activity, *sur2* mutants have phenotypes that are presumed to be associated with auxin overaccumulation (Delarue et al., 1998; Barlier et al., 2000; Bak et al., 2001). The low internal concentration of IAA derivatives we found in shoots of *cad1-3* mutants might also be related to the accumulation in indole glucosinolate (such as 4MOI3M), caused by a lack of PCS1 which hydrolyses this compound. Lastly, since tryptophan is the precursor for *de novo* biosynthesis of both IAA and indole-glucosinolates, competition for the same substrate may have concurred to cause a decrease in IAA derivatives concomitantly to an increase in 4MOI3M in the shoots of *cad1-3* mutants, even in the absence of Cd.

## Conclusion

This work highlights some novel aspects of AtPCS1 functions in *A. thaliana*. First, our study reveals the role of AtPCS1 in Cd and bacterial pathogenic resistance fully enabling callose deposition. Second, the performed analyses confirm the role of AtPCS1 in managing the glucosinolate pathway, and support the AtPCS1 involvement in the regulation of the phenylpropanoid pathway, with particular reference to the degree of stem lignification. Third, the loss of AtPCS1 suggests possible effects on the modulation of auxin content and distribution *in planta*.

## Author Contributions

MD set up the experimental plans, together with OV and LS, and performed most of the experiments. SP and EB contributed to carry out the experiments on bacterial growth assays. FG and MC executed the metabolite extraction and HPLC-ESI-MS analyses. MRC performed the measurements on lignification. AS, CB, and AA performed the auxin quali- quantification. LS and OV conceptualized the overall structure of the experimental work and critically edited it. LS and CB revised the text based on feedback from all the coauthors.

## Conflict of Interest Statement

The authors declare that the research was conducted in the absence of any commercial or financial relationships that could be construed as a potential conflict of interest.

## Footnotes

1 http://www.massbank.jp/

2 http://mzmine.sourceforge.net

## Supplementary Material

FIGURE S1 Multivariate analysis of metabolites from shoots and roots of wild-type (wt) and *PCS1* knockout mutant (*cad1-3*) grown under control conditions or after Cd exposure for 24 h. The LC-ESI-MS-detected m/z features were used as X variables. The O2PLS-DA S-loading plots p1 vs. pq(corr)1 showed the correlation of the metabolites (black squares, the q part of the model) [pq(corr), correlation between p and q] in shoots and roots with the two plant lines (the p part of the model). The models are statistically significant.

TABLE S1 List of identified metabolites from shoots and roots of wild-type and *PCS1* knockout mutant (*cad1-3*) grown under control conditions or after Cd exposure for 24 h. The analysis was performed in negative ion mode (LC-ESI-MS). The list includes the fragmentation patterns (MS/MS and MS^3^) and the putative identifications with the corresponding references. *m/z*, mass to charge ratio; rt, retention time. The MS^3^ fragmentation patterns were generated in MS/MS column by Collision Induced Dissociation (CID) of the in bold and underlined ion.

TABLE S2 List of identified auxins from shoots and roots of wild-type and *PCS1* knockout mutant (*cad1-3*) grown under control conditions. The analysis was performed in negative ion mode, except for the indole-3-acetic acid methyl ester (MeIAA), recorded in positive ion mode. Selected reaction-monitoring conditions were for protonated or deprotonated auxin metabolites ([M + H]+ or [M - H]-). *m/z*, mass to charge ratio; rt, retention time; Q1, precursor ion selected in Q1 mass filter; Q3, product ion selected in Q3 mass filter; Q2, collision energy (V).

## Abbreviations

Flg22: flagellin22
GSH: reduced glutathione
IAA: indole-3-acetic acid
IAN: indole-3-acetonitrile
IAOx: indole-3-acetaldoxime
IBA: indole-3-butyric acid
ICA: indole-3-carboxylic acid
MeIAA: indole-3-acetic acid methyl ester
PC: phytochelatin
PCS: phytochelatin synthase
*Ps*DC3000: *Pseudomonas syringae* DC3000
4MOI3M (4-methoxy-I3G in Clay et al., 2009): 4-methoxy-indol-3-ylmethylglucosinolate
4MSOB: 4-methylsulfinylbutylglucosinolate
4OHI3M: 4-hydroxy-indolyl-3-methylglucosinolate
7MSOH: 7-methylsulfinylheptylglucosinolate
8MSOO: 8-methylsulfinyloctylglucosinolate
8MTO: 8-methylthiooctylglucosinolate
