# Supplementary figures and images for: The Arabidopsis thaliana Knockout Mutant for Phytochelatin Synthase1 (cad1-3) Is Defective in Callose Deposition, Bacterial Pathogen Defense and Auxin Content, But Shows an Increased Stem Lignification
Source: Front Plant Sci. 2018 Jan 22;9:19. doi: 10.3389/fpls.2018.00019 (PMC5786554; doi:10.3389/fpls.2018.00019)

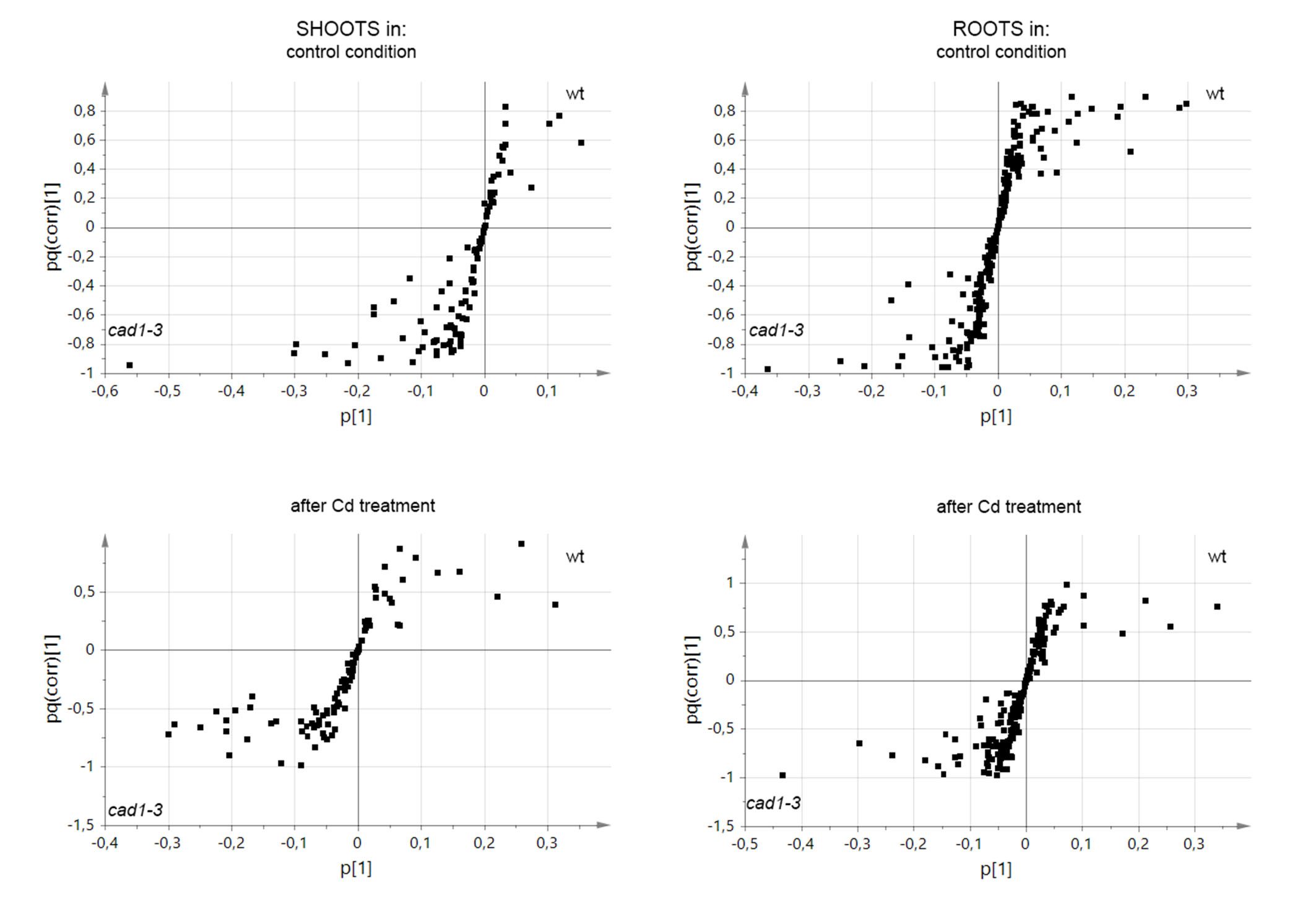

Supplement: FIGURE S1 — Multivariate analysis of metabolites from shoots and roots of wild-type (wt) and PCS1 knockout mutant (cad1-3) grown under control conditions or after Cd exposure for 24 h. The LC-ESI-MS-detected m/z features were used as X variables. The O2PLS-DA S-loading plots p1 vs. pq(corr)1 showed the correlation of the metabolites (black squares, the q part of the model) [pq(corr), correlation between p and q] in shoots and roots with the two plant lines (the p part of the model). The models are statistically significant. [file Image_1.jpg]
