# Supplementary material for: The Arabidopsis thaliana Knockout Mutant for Phytochelatin Synthase1 (cad1-3) Is Defective in Callose Deposition, Bacterial Pathogen Defense and Auxin Content, But Shows an Increased Stem Lignification
Source: Front Plant Sci. 2018 Jan 22;9:19. doi: 10.3389/fpls.2018.00019 (PMC5786554; doi:10.3389/fpls.2018.00019)
Supplement: TABLE S1 — List of identified metabolites from shoots and roots of wild-type and PCS1 knockout mutant (cad1-3) grown under control conditions or after Cd exposure for 24 h. The analysis was performed in negative ion mode (LC-ESI-MS). The list includes the fragmentation patterns (MS/MS and MS3) and the putative identifications with the corresponding references. m/z, mass to charge ratio; rt, retention time. The MS3 fragmentation patterns were generated in MS/MS column by Collision Induced Dissociation (CID) of the in bold and underlined ion. [file Table_1.docx]

| **m/z** | **rt** | **MS/MS** | **MS^3^** | **Putative identification** | **Reference** |
| --- | --- | --- | --- | --- | --- |
| 436.1 | 2.9 | m/z I %  **371.8** 100.0  258.7 6.2  373.7 4.0  194.8 2.6  176.7 2.5  177.8 2.0  420.7 1.7  144.8 1.7  257.7 1.5  128.7 1.4 | m/z I %  292.5 100.0 | 4-methylsulfinylbutyl  glucosinolate (4MSOB) | Rochfort et al., 2008 |
| 463 | 4.8 | m/z I %  **284.6** 100.0  266.7 80.7  239.7 22.9  159.7 15.0 | m/z I %  96.7 100.0 | 4-hydroxy-indolyl-3-methyl  glucosinolate (4OHI3M) | Rochfort et al., 2008 |
| 478.2 | 6.1 | m/z I %  **413.8**  100.0  414.5 13.3  415.8 3.2  462.6 1.8  194.8 1.6  290.7 1.2  463.0 1.2  258.7 1.2 | m/z I %  258.6 100.0  363.8 41.4 | 7-methylsulfinylheptyl  glucosinolate (7MSOH) | Beekwilder et al., 2008 |
| 492.2 | 13.1 | m/z I %  **427.8** 100.0  428.6 16.2  258.7 4.4  429.9 3.2  429.7 2.7  233.8 2.3  138.6 1.4  430.3 1.4  249.8 1.3  274.7 1.3 | m/z I %  258.6 100.0  194.7 70.8  226.6 67.1  232.0 66.0 | 8-methylsulfinyloctyl  glucosinolate (8MSOO) | Glauser et al., 2012 |
| 477.1 | 15.8 | m/z I %  **258.6** 100.0  274.7 69.9 | m/z I %  96.7 100.0 | 4-methoxy-indol-3-ylmethyl  glucosinolate (4MOI3M) | Rochfort et al., 2008 |
| 385.1 | 15.2 | m/z I %  **222.7** 100.0  204.7 72.4  246.7 49.3  163.8 15.7  205.8 10.4  231.7 8.9  189.7 8.0  223.7 7.2  247.7 6.8  264.7 4.8  366.7 3.3  148.8 2.6  152.8 2.3  294.7 1.9 | m/z I %  163.7 100.0  207.7 32.9  178.7 17.4  220.7 14.3  148.7 11.6  164.6 10.3 | sinapic acid hexoside | Stehle et al., 2008 |
| 551.1 | 17 | m/z I %  **388.8** 100.0  192.7 46.6  389.6 8.7  194.7 7.7  340.8 6.7 | m/z I %  192.7 100.0 | ferulic acid derivative | Mena et al., 2016 |
| 609 | 20.5 | m/z I %  **446.8** 100.0  300.8 93.7  462.7 47.5  445.9 17.1  447.9 11.6  301.9 11.4 | m/z I %  300.7 100.0  298.7 25.7 | quercetin hexoside  rhamnoside | Routaboul et al., 2006 |
| 577.2 | 25.5 | m/z I %  284.8 100.0  **430.8** 95.8  431.4 15.5  285.6 13.8  430.1 4.4  308.9 1.9  212.9 1.0  241.8 1.0 | m/z I %  284.7 100.0  285.7 10.7  283.9 6.6  282.9 2.8  326.7 2.6  254.8 1.8  226.7 1.7  242.8 1.2  152.7 1.1 | kaempferol 3,7  dirhamnoside | Routaboul et al., 2006 |
|  |  |  |  |  |  |
| Metabolites were ionized in negative modality (LC-ESI-MS) | | | | |  |
| The ion in bold and underlined was that selected for the next fragmentation | | | | |  |
