# Supplementary material for: The Arabidopsis thaliana Knockout Mutant for Phytochelatin Synthase1 (cad1-3) Is Defective in Callose Deposition, Bacterial Pathogen Defense and Auxin Content, But Shows an Increased Stem Lignification
Source: Front Plant Sci. 2018 Jan 22;9:19. doi: 10.3389/fpls.2018.00019 (PMC5786554; doi:10.3389/fpls.2018.00019)
Supplement: Supplementary file 3 [file Table_2.docx]

rt	Q1 (m/z)	Q3 (m/z)	Q2 (V)	Putative identification
5.06	160.0	115.8	–22	indole-3-carboxylic acid (ICA)
6.29	174.0	129.6	–14	indole-3-acetic acid (IAA)
11.35	202.0	157.5	–16	indole-3-butyric acid (IBA)
9.58	190.1	129.9	17	indole-3-acetic acid methyl ester (MeIAA)
